# The Effect of a Healthy Lifestyle Intervention on Shift Workers With Acid Peptic Disease: A Quasi-experimental Study in Karaikal, South India

**DOI:** 10.7759/cureus.67642

**Published:** 2024-08-23

**Authors:** K. Murugesan, K. Mujibur Rahman, R. Niranjjan, S. Sathish Kumar, Nancy Sebastian

**Affiliations:** 1 Preventive Medicine, Vinayaka Mission’s Medical College and Hospital, Karaikal, IND; 2 Epidemiology and Public Health, Otorhinolaryngology, Vinayaka Mission’s Medical College and Hospital, Karaikal, IND; 3 Preventive Medicine and Biostatistics, Vinayaka Mission’s Medical College and Hospital, Karaikal, IND; 4 Preventive Medicine and Community Medicine, Vinayaka Mission's Medical College and Hospital, Karaikal, IND

**Keywords:** quasi-experimental design, food habits, acid peptic disease, healthy lifestyle, shift workers

## Abstract

Acid peptic disease (APD) is the most common health issue among rotational shift workers, including night shifts. Alterations in diet patterns from the routines of life play a key role in the development of APD in this population. This study aims to determine food habits and evaluate the effect of a healthy lifestyle intervention on reducing the frequency of the occurrence of APD among rotational shift workers. A quasi-experimental study was carried out for six months in Karaikal, South India. About 37 employees involved in rotational shift work were recruited consecutively. A healthy lifestyle intervention was implemented, and pre and postintervention data were collected using a pre-tested, semi-structured questionnaire.

The McNemar test was employed to assess the pre and postintervention data using SPSS Statistics version 24 (IBM Corp., Armonk, NY, USA). Among 37 shift workers, almost 21 (56.8%) were suffering from heartburn, and about 14 (38%) presented with two symptoms. Before the intervention, 24 (64.9%) workers experienced APD > 28 days after the previous episode. After intervention, a majority of 34 (91.9%) workers experienced APD > 28 days after the previous episode (p-value = 0.002). This highlights the importance of healthy lifestyle modifications in controlling APD among shift workers. This in turn improves the productivity of the workers.

## Introduction

Globalization is one of the most essential concepts for creating new business prospects for the younger generation as well as developing industries that may offer good sales beyond regional boundaries [1]. It refers to a market size that is not severely limited by geographical boundaries or other causes. The market is the finest alternative for product creators and manufacturers to sell their products to increase industry demand [1,2]. In this scenario, several industries have altered their work schedules to match the needs and functions that occur outside of normal business hours [3-5]. Industries offer attractive compensation to entice people to work constantly on a shift basis (including night shift), which causes humans to change their lifestyle and develop stomach ulcers and other health disorders [6-11].

Health-related concerns among workers not only harm the individual but also the company that employs him or her because the individual's absence disrupts the flow of work, perhaps causing a delay or halt of the production unit [4]. As a result, this topic raises awareness among individuals to prevent health issues in shift workers. Some common health issues that have been addressed among shift workers for a long time include acid peptic disease (APD), dyslipidemia, myocardial dysfunction, hypertension, diabetes mellitus, psychological stress, and depression as a result of lifestyle changes and changes in the biological clock and sleep pattern [12-19].

Steps have been attempted to prevent or manage regularization or bridge the lifestyle to minimize the deviation from normal, which can contribute to reducing or preventing health difficulties from occurring or worsening due to shift work [20]. There is a significant gap when shift workers deviate from their typical lifestyle, and therefore, they should be taught about both normal and healthy lifestyles [14,21]. Many countries, even developing ones, have seen an increase in the number of shift workers. A healthy lifestyle allows one to have a more sophisticated or better life in terms of physical, mental, and financial health [15]. Hence, the present study was executed to determine the food habits and evaluate the effect of a healthy lifestyle intervention in reducing the frequency of occurrence of APD among rotational shift workers.

## Materials and methods

A quasi-experimental (pre and postintervention) study was carried out among rotational shift workers, including those on night shifts, for six months (from January to June 2023). The study was approved by the Vinayaka Mission's Research Foundation Ethics Committee (approval no. VMMC/CM/2023/114). About 55 subjects were recruited from different organizations in Karaikal; all of them worked on a shift basis for eight to 12 hours per day. Workers who attended the hospital OPD were selected consecutively during the study period. Subjects working in any industry in a rotational shift pattern and with known cases of APD were included. Volunteers with the habit of smoking, alcoholism, excessive stress, non-responders, and others with gastrointestinal disorders, any other kind of disorder that may interfere with or mimic gastrointestinal symptoms, myocardial dysfunction, hepatitis, etc., were excluded from the present study [7-10]. Out of 55 employees, 19 subjects were excluded based on the exclusion criteria.

Data collection

Preintervention data was collected for all samples prior to the lifestyle modification suggestions and was recorded once again 48 days after following the lifestyle modification. The preassessment questionnaire contains 15 prevalent parameters: stomach pain, heartburn, chest pain, nausea, vomiting, bloody vomiting, regurgitation, trouble breathing, cough, feeling of fullness, bloating, indigestion, appetite changes, frequent defecation, bloody or dark stools, and referred pain. The severity of APD recorded during the four weeks was measured by a 5-point Likert scale (0-5) from 'never' to 'severe.' Any subject with at least one or more complaints in the past four weeks was considered a patient with a positive history of APD. Answers to other questions, such as tea and coffee consumption and stress status, were also collected. Upper gastrointestinal diseases were defined as positive if reflux, acute or former gastritis, or acute, former, or chronic ulcers were reported in the medical history or questionnaire. We also documented any therapies with antacid medication, eradication, or operation. The postintervention questionnaire concentrated on whether the shift workers adopted healthy lifestyle modifications or not. In addition, altered food patterns and improvements in the severity of the symptoms were assessed and compared with the preintervention data.

Intervention

A healthy lifestyle intervention was implemented among rotational shift workers. The participants were advised not to skip any food, i.e., breakfast, lunch, or dinner; to maintain an average sleep hour of six to eight hours; to nap for 20 to 30 minutes if work permits; to restrict the duration between two major meals to less than six hours and snack in between if the duration between meals is more than six hours; to avoid or reduce intake of tea and coffee and opt for milk instead; and to avoid lying down immediately after food for at least 45 minutes. These healthy lifestyle alterations were suggested after analysis of the study participants and their deviations from the routine with individualized suggestions. The post-lifestyle modification assessment was done after an interval of 48 days.

Statistical analysis

All statistical analyses were performed using SPSS Statistics version 24.0 (IBM Corp., Armonk, NY, USA). Data were presented as mean and standard deviation for continuous variables and frequency and percentages for categorical variables. The McNemar test was used to analyze the difference between pre and postintervention data. A p-value of < 0.05 was considered a statistically significant difference. The graphs were constructed using GraphPad Prism 5 (GraphPad Software, La Jolla, CA, USA), and the data organization was done with Microsoft Excel (Microsoft Corp., Redmond, WA, USA).

## Results

Occurrence of symptoms among shift workers

**Figure 1:**
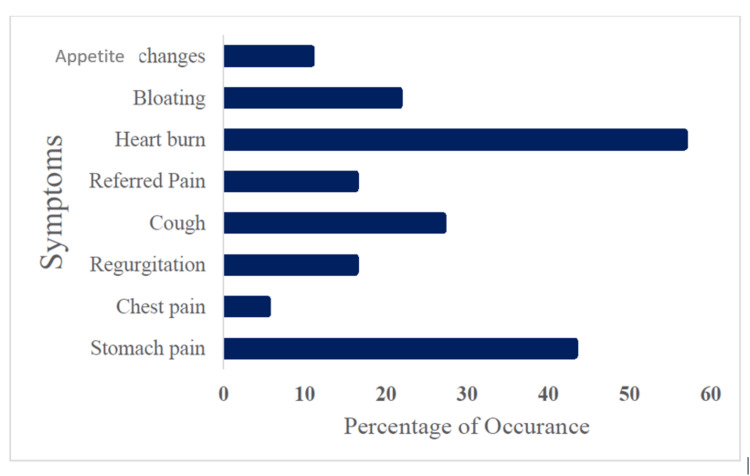
Percentage of occurrence of symptoms among shift workers (n = 37) Image credit: Murugesan K, Rahman KM, Niranjjan R, et al.

In the present study, the age of the subjects ranged from 22 to 50 years, with a mean age of 36.51 ± 6.55 years. Among 37 subjects, 30 (81%) were males and seven were females. Among the listed symptoms, the majority of the participants had heartburn as their presenting complaint, followed by other symptoms. Among 37 shift workers, almost 21 (56.8%) were suffering from heartburn, 16 (43.2%) had stomach pain, 10 (27%) experienced cough, eight (21.6%) presented with bloating and fullness, six (16.2%) had referred pain, six (16.2%) had regurgitation, four (10.8%) experienced appetite changes, and two (5.4%) suffered from chest pain (Figure 1).

Notably, many presented with more than one symptom among the listed 15 symptoms. It was observed that 14 (38%) of the individuals presented with two symptoms (di-symptomatic on the preassessment), while 12 (32%) presented with one symptom (monosymptomatic), and the remaining 11 (30%) individuals presented with more than two symptoms (polysymptomatic).

The duration of the disease was noted in a wide range, from a minimum of one year to a maximum of 15 years. It was found that there was an equal number of members (17 each) in the range of one to six years and six to 11 years, and three members in the range of 11 to 16 years, with a mean of 7.11 ± 3.11 years. Interestingly, it was found that of 37 participants, 15 (41%) skipped lunch during night shift work, and 22 (59%) participants had not skipped lunch. Five (14%) workers were noted to be taking midnight snacks.

Average sleep duration in night shift, day shift, and off-duty period

**Figure 2:**
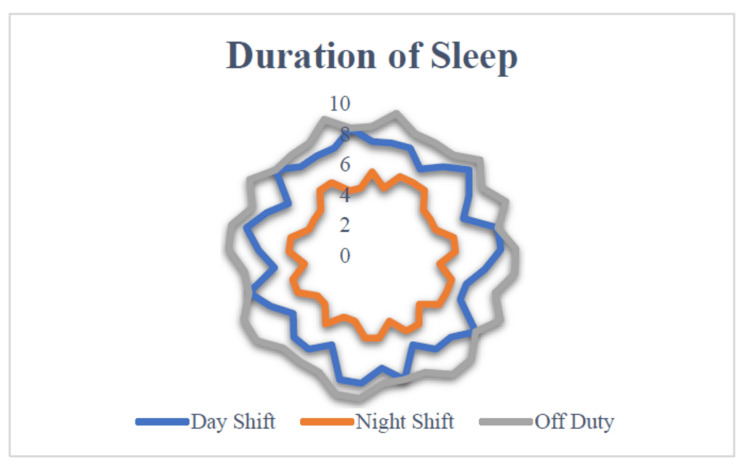
Average sleep duration during the night shift, day shift, and off-duty period (n = 37) Image credit: Murugesan K, Rahman KM, Niranjjan R, et al.

There was a marked difference in the average sleep hours during the night shift compared to the day shift and off-duty periods. The average sleep duration during the night shift ranged from four to six hours. In the day shift, the average sleep duration was found to be six to eight hours, while the same average sleep duration during the off-duty period was in the maximum range of eight to 10 hours (Figure 2).

Comparison of duration between APD episodes before and after intervention

**Figure 3:**
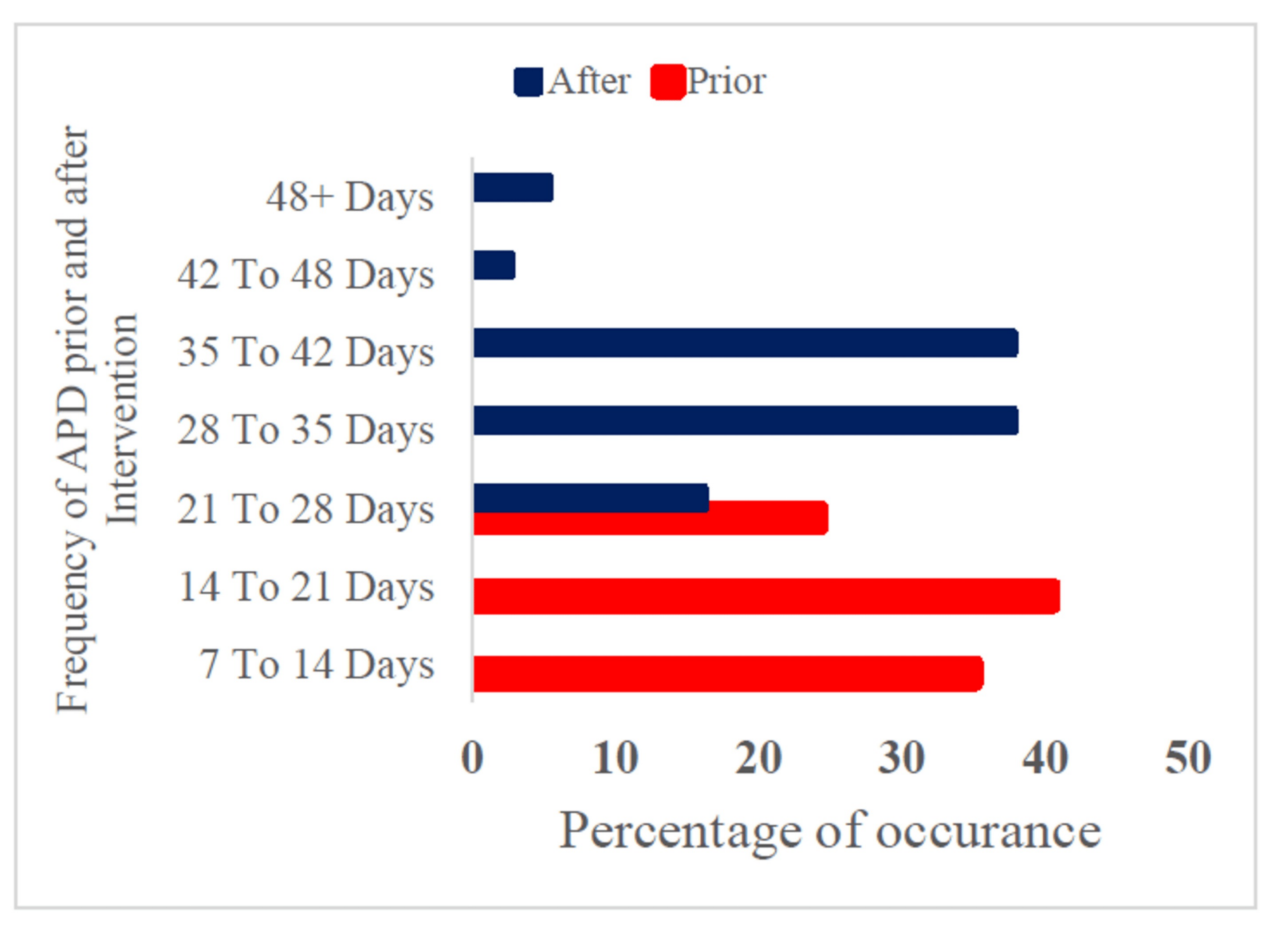
Comparison of duration between APD episodes before and after Intervention (n = 37) APD: Acid peptic disease Image credit: Murugesan K, Rahman KM, Niranjjan R, et al.

Before the intervention, the frequency of the episodes of acid peptic disease ranged from 10 to 28 days. Of the participants, 13 (35.1%) fell under the 10 to 14-day range, 16 (43.2%) were between 14 and 21 days, and eight (21.6%) were under the 21 to 28-day range. After intervention, the duration taken for the next episode to occur ranged from 26 days to more than 48 days. Notably, the average days between two episodes before intervention were 14 days, and the average days taken for the next episode to occur after intervention went up to 34 days (Figure 3).

Table 1 shows that before the intervention, about 24 (64.9%) shift workers experienced APD > 28 days after the previous episode, and after the intervention, a majority of 34 (91.9%) experienced APD > 28 days after the previous episode. Notably, a 27% improvement in the occurrence of APD symptoms after intervention was statistically significant (chi-square (c^2^) = 9.38; degree of freedom (df) = 1; p = 0.002).

**Table 1:** Comparison of duration between APD episodes before and after intervention (n = 37) *McNemar value, **p-value < 0.05 APD: Acid peptic disease, c^2^: Chi-square, df: degree of freedom

Duration between APD episodes	Preintervention n (%)	Postintervention n(%)	c^2^; df; p-value
< 28 days	13 (35.1)	3 (8.1)	9.38*; 1; 0.002**
> 28 days	24 (64.9)	34 (91.9)	

## Discussion

In the current study, the majority of the participants had heartburn as their presenting complaint, followed by other symptoms. Many presented with more than one symptom among the 15 listed symptoms. The duration of the disease was noted to range widely from a minimum of one year to a maximum of 15 years. About 41% and 86% of the workers skipped lunch and snacks during night shifts, respectively. There was a marked difference in the average sleep hours during the night shift compared to the day shift and off-duty periods. The average days between two episodes prior to intervention were 14 days, and the average days taken for the next episode to occur after intervention went up to 34 days. Notably, healthy lifestyle interventions demonstrated a 27% improvement in the occurrence of APD symptoms after intervention.

Acid peptic disorders include a variety of illnesses that arise from damages caused by acid and peptic action in gastric secretions, including gastroesophageal reflux disease (GERD) and peptic ulcer disease, the two most severe and well-defined disease states [18]. In this study, we found that the age and duration of APDs had a high correlation. It is more prevalent in males than females. In the present study, nearly 56.35% recorded that heartburn was the priority symptom, followed by stomach pain for 43.5% and cough for 27.6% of those who worked the night shift. Malfertheiner et al. state that the predominant symptom of uncomplicated APD is epigastric pain or heartburn [22]. Similarly, our study also showed that heartburn was the most common presenting complaint.

There is a higher incidence of indigestion and peptic ulcers among rotating shift workers than among fixed, day shift workers. Many researchers have shown that there is enough evidence of health-related complications that arise during shift work and that this may not be the direct effect of the work [12-14, 22]. The prevalence of GI symptoms was significantly higher in rotating shifts than in day shifts due to irregular meal consumption among the rotating shift workers [15, 16]. However, shift work, including night shifts, is well documented to indicate that there is an increase in risk factors for health that interfere with biological functions and social life due to a mismatch between the person's circadian rhythm and environmental synchronizers [14]. This mismatch has a negative influence on performance, accidents, social responsibilities, the care of children, sleep disorders, neurophysiological problems, digestive and cardiovascular diseases, and reproductive issues [20].

As per the International Classifications of Sleep Disorders (ICSD) defined by the American Academy of Sleep Medicine (AASM, 2014), shift workers are at increased risk for a wide range of illnesses [23]. The preassessment of workers showed that there was a lack of knowledge about lifestyle and its importance, which could help them prevent health issues and maintain a good and healthy life [3]. Only a handful of studies have spoken about the lifestyle and its impact on the shift worker's health issues.

Paula et al. mentioned in their study that the gastrointestinal tract is persistently exposed to our day-to-day dietary changes, and on recognizing this, they proposed diet therapy as an integral part of the management of *Helicobacter pylori *infection in APD and as a way to a healthy life. They found that there was a strong correlation between dietary and lifestyle habits and the occurrence of pathogens. They concluded by saying that a proper nutritious diet, including fruits, vegetables, and probiotics, and avoiding certain foods such as traditional salt and preserved meat and habits such as smoking, alcohol, etc., helped in controlling the consequences and eradicating *H. pylori* infection [24].

Raiha et al., in a nationwide twin cohort study, analyzed the contribution of genetic and environmental factors in the pathogenesis of APD. In this study, they found that environmental factors were the major cause of APD, and genetic factors were modest [25]. Rutenfranz mentions that for night shift workers, there was a reduction in sleep during the night shift, with an average sleep of four to six hours. Likewise, our study also shows a similar reduction. Further, he added that this sleep reduction was possibly due to daytime sleeping, which was unfavorable and not running parallel with the circadian rhythm [26].

In our study, the sample size was smaller because we included only those workers who reported to the hospital consecutively. Further, the response to the modifications was noted in a short period of 48 days; to have more precise data, long-term follow-up is required. This study was carried out in and around Karaikal only, and the same can be carried out in multiple centers. Errors due to self-reported findings can occur. Non-response was minimal owing to the good rapport with the workers.

## Conclusions

Providing good lifestyle suggestions and interventions would benefit shift workers physically, psychologically, and economically. In this study, it was noted that there was minimal awareness among shift workers about the health issues that can arise over time because of lifestyle alterations. It was also evident that there was a significant impact on the disease by following a better lifestyle. Therefore, while considering a job that involves rotational shifts, including night shifts, the importance of a good lifestyle and how to minimize deviations from a normal lifestyle should be taken care of. Further, this improves the productivity of the workers in the long run. Thus, the importance of a healthy lifestyle and its impact on the well-being of the employee should be added as a part of occupational health training.
